# Cemento-Ossifying Fibroma of the Mandible: A Case Report

**DOI:** 10.7759/cureus.55063

**Published:** 2024-02-27

**Authors:** Hamza Salema, Vivek S Nair, Vikrant Sane, Nishita Bhosale, Rucha D

**Affiliations:** 1 Oral and Maxillofacial Surgery, Bharati Vidyapeeth Dental College and Hospital, Pune, IND; 2 Oral and Maxillofacial Surgery, Bharati Vidyapeeth Dental College and Hospital, pune, IND; 3 Periodontology, Bharati Vidyapeeth Dental College and Hospital, Pune, IND; 4 Pedodontics, Bharati Vidyapeeth Dental College and Hospital, Pune, IND

**Keywords:** excision, cementum, psammoma bodies, expansile lesion, cemento-ossifying fibroma

## Abstract

Benign osseous tumors of mesodermal origin that are included within the group of fibro-osseous lesions include cemento-ossifying fibromas (COFs). The fibrocellular component of these diseases originates from the periodontal ligament, which deposits bone and cementum encased in fibrous tissue. It typically appears in the mandible and presents as a solitary, nonaggressive, slowly developing, asymptomatic, expansile lesion, rarely occurring in the maxilla.

The only intervention that proved to be successful in producing excellent outcomes and that may be regarded as a final therapeutic option is the complete surgical removal of COFs. Presenting herein is a case report describing a painless and expansile mass in the left mandibular region, histopathologically diagnosed as COF.

## Introduction

In 1992, the World Health Organization (WHO) classified fibro-osseous neoplasms into four distinct types: fibrous dysplasia, ossifying fibroma (OF), cemento-ossifying fibromas (COFs), formerly known as cementomas, and cementifying fibroma [1].

These neoplasms are typically observed in middle-aged females, predominantly in the mandible, especially in the premolar-molar area [2]. There are two types of COF: conventional COF and juvenile COF. Conventional COF presents as slow-growing, while juvenile COF grows quickly [3]. It is thought to originate from multipotent mesenchymal cells in the periodontal ligament area, which can create fibrous tissue, cementum, and lamellar bone [4,5].

COFs are intra-bony masses that are often benign, characterized by slow growth, well-demarcation, encapsulation, and asymptomatic presentation. Over time, they may induce face distortion through expansion, requiring surgical intervention [1-3]. When viewed radiologically, COF appears as a distinct lesion that is either unilocular or multilocular, has smooth outlines, and varies in radiolucency depending on the lesion's maturity [6]. The histological characteristics of COF usually comprise storiformly arranged, multiplied fibroblasts that generate collagen fibers along with cementum granules and bone spherules in the stroma, which appear as *psammoma-like* entities [7]. Although surgery is the treatment of choice for COF, injectional steroid therapy can also be considered a conservative treatment option in certain cases.

The following case report details a painless, expansile, asymptomatic swelling in a 47-year-old male who presented to the OPD. The swelling was surgically excised and histopathologically diagnosed as COF. The differential diagnosis of the lesion includes fibrous dysplasia and cementifying fibroma.

## Case presentation

**Figure 1:**
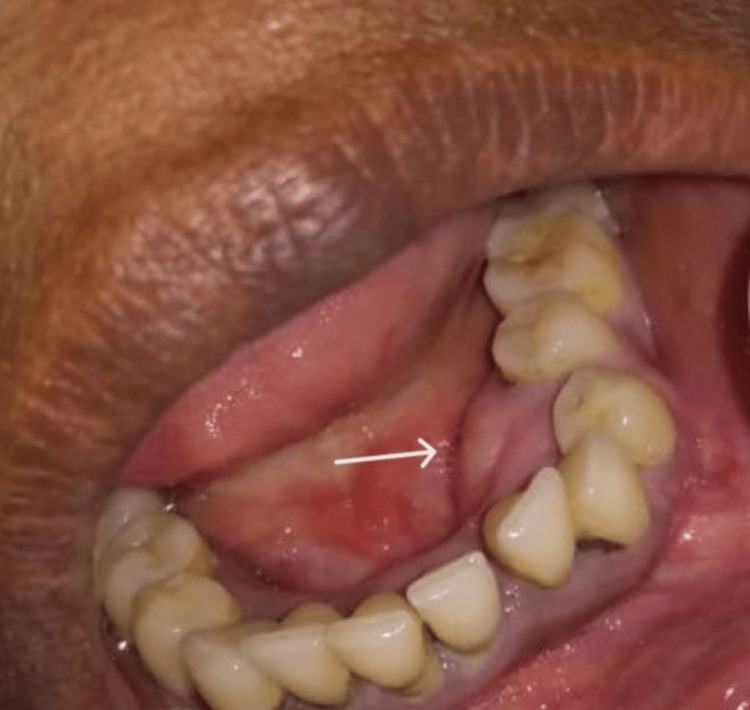
Painless spherical expansile mass observed in the mandibular lingual premolar region between teeth 33 and 35, as per FDI notation. FDI, Fédération Dentaire Internationale

The 47-year-old male patient who presented to the outpatient department had progressive swelling *with no medical history*, as detailed in the following sections. The patient reported having a gradual swelling in the left mandibular *lingual *region (Figure 1). 

**Figure 2:**
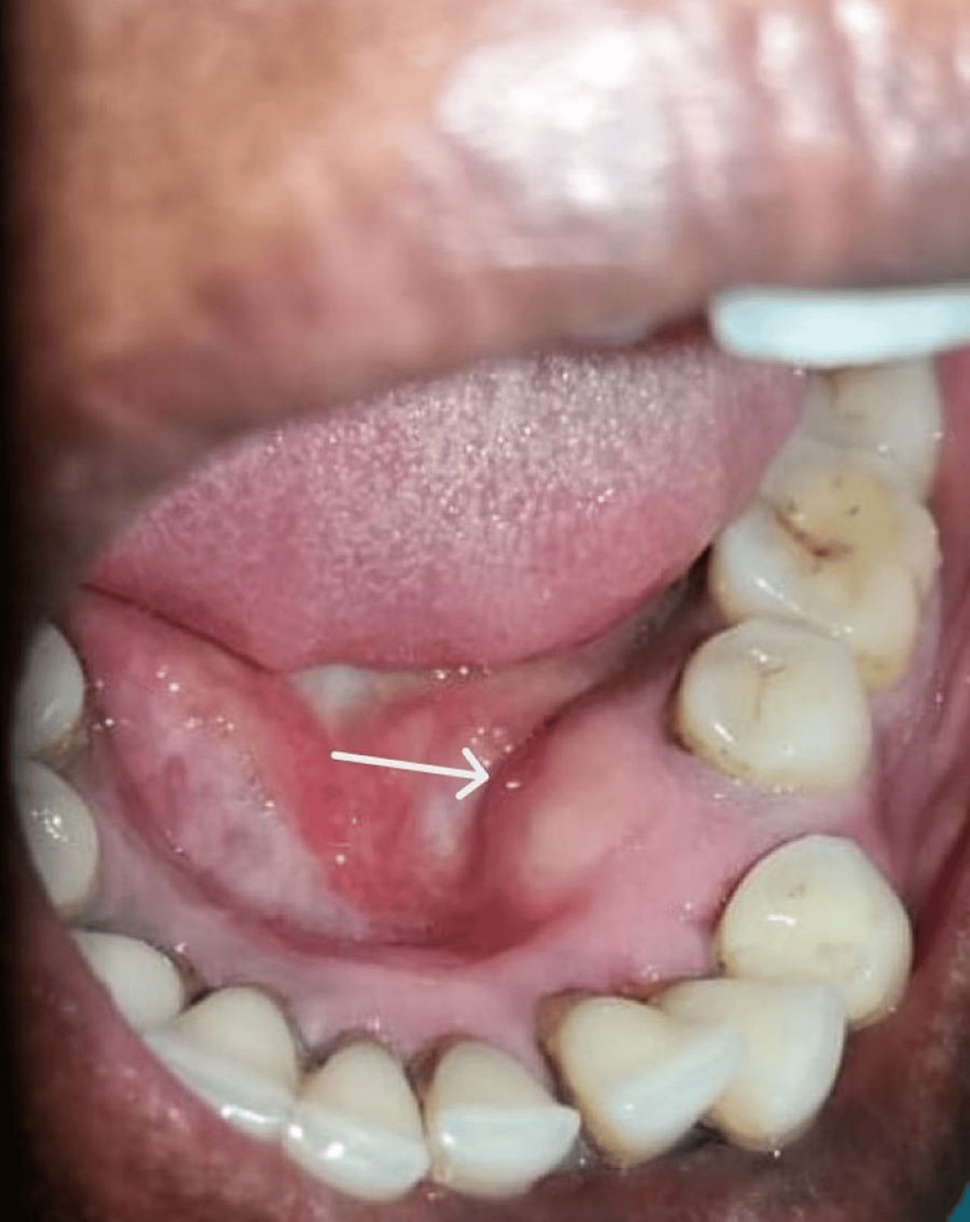
Lingual cortical expansion observed in the mandibular premolar region.

Upon examination, a single, firm, well-defined, pinkish, spherical expansile lesion was found in the left mandibular body region. It was asymptomatic and nontender. The only other complaint was that the lesion was expansile, causing significant facial asymmetry and mobile teeth. Intra-orally, teeth 33-35 showed signs of grade II mobility, and the same area showed clearly defined swelling, with the expansion of the lingual and buccal cortices. There were no signs of inflammation, and the mucosa covering the lesion was nonresilient (Figure 2).

**Figure 3:**
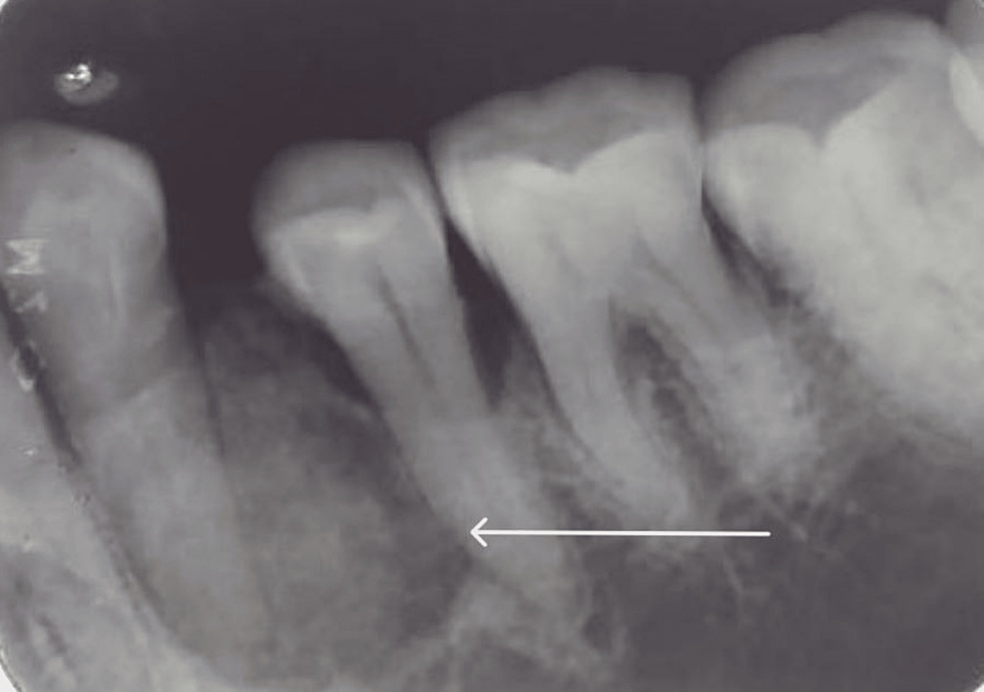
IOPA revealing a unilocular mixed lesion with an internal ground-glass appearance, a thin radiopaque margin, and concentric expansion of the affected bone involving teeth 34-35. IOPA, Intraoral Periapical Radiograph

The affected area required an intraoral periapical X-ray, which revealed a radio-opaque lesion with clearly defined borders. Between teeth 34 and 35, there were centrifugally growing calcific flecks visible. Following routine blood investigations, a biopsy of the affected region was carried out that suggested COF (Figure 3).

**Figure 4:**
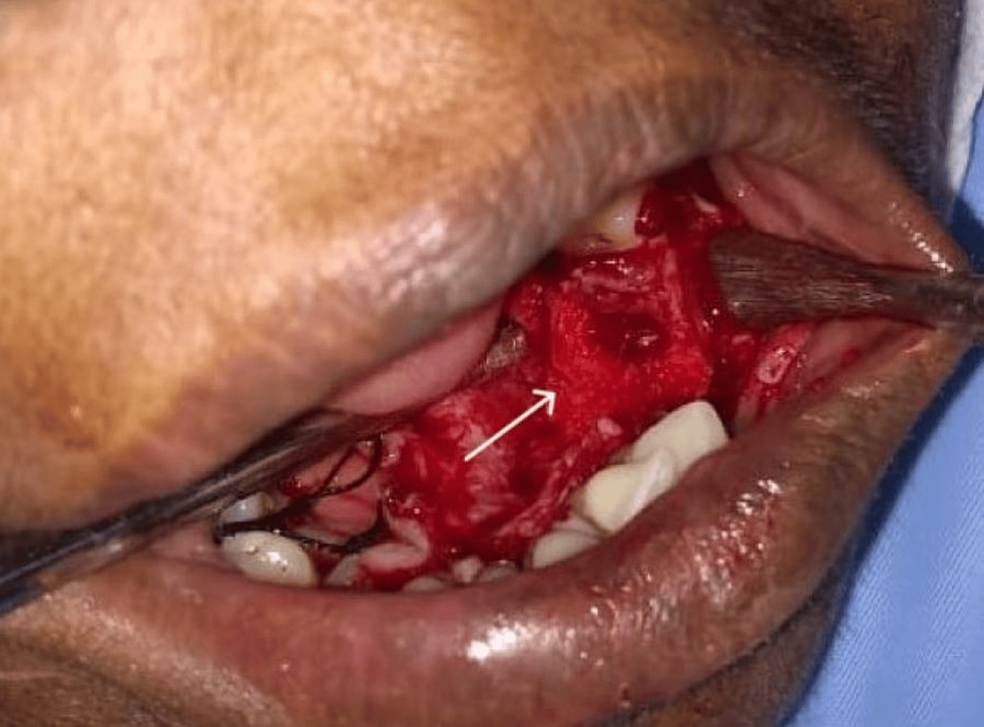
Mucoperiosteal flap elevated followed by curettage in the premolar region, exposing the lesion along with the extraction of teeth 34 and 35.

**Figure 5:**
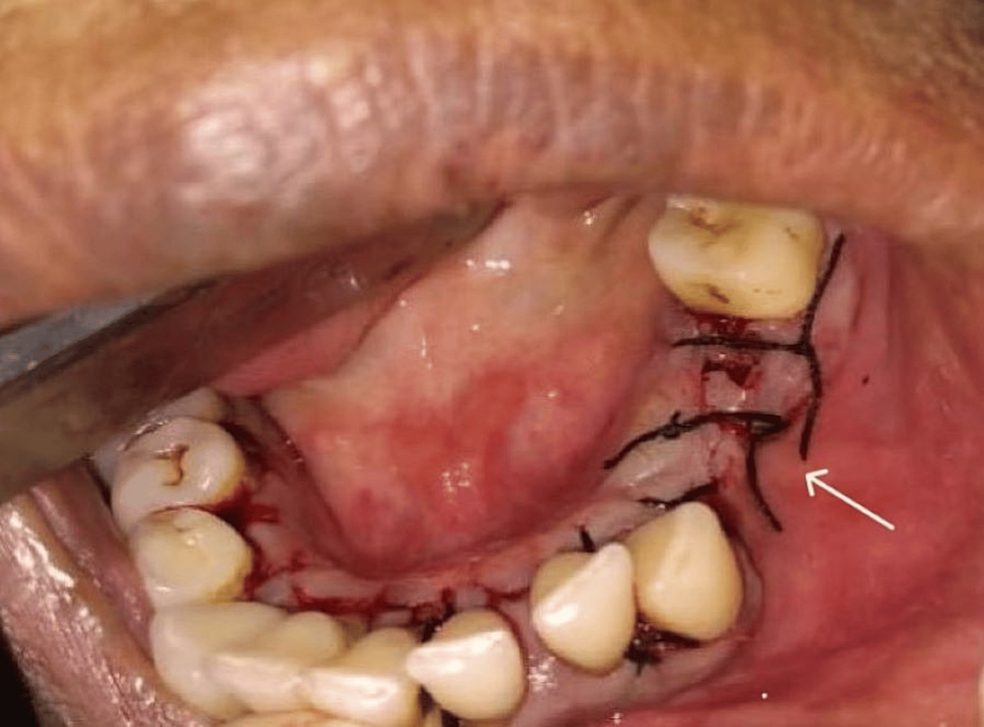
Primary closure achieved with simple interrupted sutures with silk 3-0.

Depending on the results of the histology and radiography, surgical excision of the lesion was carried out under preoperative antibiotic coverage, and postoperatively, antibiotics and analgesics were prescribed. In the affected area, a mucoperiosteal flap was elevated following the extraction of teeth 34 and 35. The lesion was excised in its entirety, followed by curettage of the area. The flap was closed using 3-0 black braided silk interrupted sutures, and the patient was scheduled for a follow-up appointment one week later for suture removal. The patient was regularly followed up for three months postoperatively (Figures 4-5).

**Figure 6:**
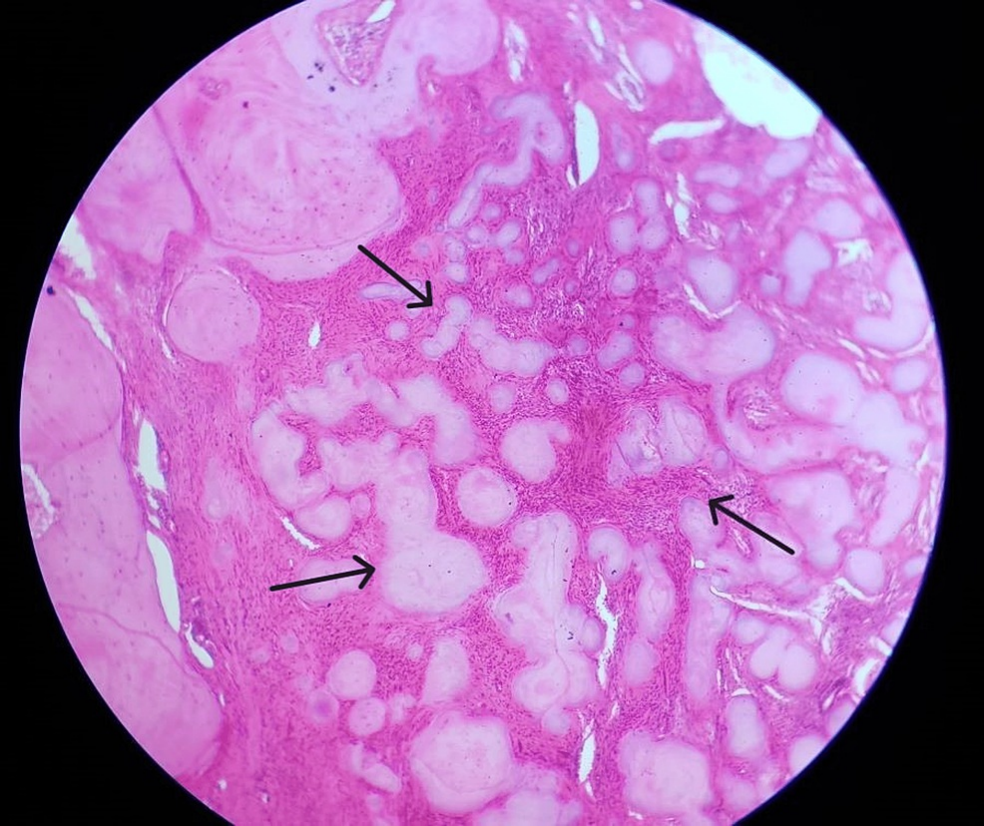
The histopathological specimen presented with a highly cellular fibroblast field with dispersed areas of calcifications, similar to cementum-like material.

The histopathological specimen was observed and presented with a highly cellular fibroblast field with dispersed areas of calcifications. The calcifications were similar to cementum-like material. This was suggestive of COFs (Figure 6).

## Discussion

In 1992, WHO distinguished between four distinct types of fibro-osseous neoplasms, which were previously known as cementomas: fibrous dysplasia, OF, COFs, and cementifying fibroma [1]. At the WHO Head and Neck Consensus Conference in 2003, under the heading *Neoplasms and other lesions occurring in the maxillofacial skeleton*, osseous neoplasms and nonneoplastic lesions were classified, with COF assigned to the neoplastic lesions [6].

COF undergoes membranous ossification and is predominantly observed in the maxillofacial bones, most commonly in the mandible, followed by the maxilla [1,8]. Although they sporadically appear in the paranasal sinus, temporal bone, nasal bone, and orbital bone, these lesions are most common in the premolar-molar region of the mandible. When it affects the nasal bone or the orbital bone, it can cause symptoms like purulent rhinorrhea and orbital displacement [3]. According to a study by Sui et al., the lesion primarily affects women and typically manifests in their second or fourth decade of life [2].

Various etiological factors, such as extraction or trauma, can cause COF in the bone to form cementum, bone, fibrous tissue, or a combination of these elements. It is thought that COF originates from the mesenchymal blast cells of the periodontal ligament [2]. A metaplastic process that emerges in the connective tissue fibers and unregulated growth of periodontal ligament cells are the two potential sources of the large amounts of mesenchymal cellular induction into bone and cementum that result in odontogenesis [3]. According to Dewan et al.'s hypotheses, the periodontal membrane may be stimulated by infection-induced inflammation and fibrosis of the periapical region [9].

COFs are intra-bony masses, ranging from spherical to egg-shaped, and can be observed as solitary lesions or rarely in multiple sites and show multiple, familial, or sequential presentations [5]. They are often benign, but in rare cases, they may present as a central variant, which tends to induce neoplastic changes [10]. Although it grows slowly, it can occasionally appear as juvenile aggressive ossifying fibroma (JAF), a form of OF that manifests more vascularity and aggression in people aged between 5 and 15 years [3]. Overall, COF is well-defined, encapsulated, and asymptomatic and may eventually cause facial distortion as a result of expansion, requiring surgical intervention [9,11]. Teeth displacement may be the only clinical feature of the ensuing facial asymmetry. Seldom does root resorption occur; teeth close to the lesion nevertheless remain vital [2]. Typically, growth arises concentrically apically to the molars and premolars above the mandibular canal. Both the skin or mucosa covering the bone and its cortical bone plates maintain their integrity intact [4].

Depending on the degree of mineralization, COF presents with a variety of radiographic patterns. It is more common in younger patients and appears radiolucent in the early stages. As the lesion matures, calcific flecks become more numerous and eventually progress to a complete radiopaque mass, which is frequently seen in older patients [2]. COFs typically have an expansile mass that is round or oval in shape, smooth, well-defined, and bordered by cortices. They grow centrifugally, expanding uniformly in all directions to give the appearance of a well-defined round mass [4]. In a small number of cases, additional radiological characteristics like root resorption, tooth displacement, and erosion of the mandible's inferior border may also be observed.

A well-defined unilocular mixed lesion with an internal ground-glass appearance and a thin radiopaque margin is visible on an orthopantomogram (OPG); root resorption may be present. An expansion of the affected bone may be seen in a concentric corticated lesion with radiopaque foci on a coronal cone-beam computed tomography (CBCT). Typically, COF presents on a CT scan as an expansile, well-circumscribed lesion with a ground glass appearance that may include a cystic component. Precontrast T1-weighted MRI images exhibit intensities akin to brain gray matter, while T2-weighted sequences show intermediate to low intensities. Following gadolinium injection, the lesion is enhanced [4].

According to various authors, the radiographic pattern for COF is characterized as follows: As reported by Bala et al., 26% of COF cases exhibited lytic lesions, 63% had lytic lesions with radiopaque foci, and 12% displayed diffuse, homogeneous lesions [10]. The radiographic pattern was classified as follows by Barberi et al.: lesion with ill-defined border (15%), defined lesion with sclerotic rim (45%), and defined lesion without sclerotic rim (40%). Nearly 93.6% of lesions had well-defined margins that made them easily distinguishable from healthy bone, according to Kaur et al. [5].

Histologically, OF manifests as a largely avascular fibrous stroma with spheroidal calcifications that resemble cement structures and fusiform cells mixed with bone trabeculae. There might be multinucleated giant cells. The calcified material comprises trabeculae of woven bone with irregular shapes, scattered trabeculae of lamellar bone, round or oval deposits of basophilic staining, and cellular or acellular calcified deposits resembling cementum. This combination is often referred to as psammoma bodies. COF has a cellular connective tissue with mineralized material and osteoblastic rimming visible on the surface under a microscope [4].

The recommended courses of treatment for COF include surgical removal, curettage, and excision. While moderately large lesions require aggressive treatment, such as local excision or curettage when appropriate, smaller lesions can be treated with enucleation and primary closure. When a radiographic examination reveals no cleavage margin, the lesion is completely enucleated and treated as a smaller lesion. Due to the tendency for recurrence following incomplete removal, larger COFs require a more drastic approach. In these situations, mono-bloc resection should be carried out in conjunction with bony reconstruction, using a free fibula flap or an iliac crest nonvascularized bone graft, depending on the size and volume of the defect. Radiotherapy is strongly discouraged because of the lesions' radioresistance and the likelihood of radiation-induced sarcomas [5].

## Conclusions

When tumors are radiographically well-circumscribed, contain material that resembles cementum and bone, and may or may not have psammoma-like bodies, a diagnosis of COF is made.

It is uncommon for the maxilla and mandible to exhibit synchronous OF at several sites. In most OF cases, surgical excision is sufficient, but radical surgical resection is indicated for extensive lesions. For large lesions, extensive en bloc resection is recommended to lower the chance of recurrence.

## References

[1] Al-Shaham AA, Samher AA (2010). Cemento-ossifying fibroma of the maxilla. J Plast Surg Hand Surg.

[2] Katti G, Khan MM, Chaubey SS, Amena M (2016). Cemento-ossifying fibroma of the jaw. BMJ Case Rep.

[3] Bhat SV, Kumar SP, Periasamy S, Krishna VK (2022). An uncommon presentation of ossifying fibroma in the maxilla. Cureus.

[4] Chidzonga M, Sunhwa E, Makunike-Mutasa R (2023). Ossifying fibroma in the maxilla and mandible: a case report with a brief literature review. Cureus.

[5] Kaur T, Dhawan A, Bhullar RS, Gupta S (2021). Cemento-Ossifying Fibroma in Maxillofacial Region: A Series of 16 Cases. J Maxillofac Oral Surg.

[6] Mohapatra M, Banushree CS, Nagarajan K, Pati D (2015). Cemento-ossifying fibroma of mandible: an unusual case report and review of literature. J Oral Maxillofac Pathol.

[7] Sopta J, Dražić R, Tulić G, Mijucić V, Tepavčević Z (2011). Cemento-ossifying fibroma of jaws-correlation of clinical and pathological findings. Clin Oral Investig.

[8] Trijolet JP, Parmentier J, Sury F, Goga D, Mejean N, Laure B (2011). Cemento-ossifying fibroma of the mandible. Eur Ann Otorhinolaryngol Head Neck Dis.

[9] Dewan HS, Dewan SK, Bahl S, Tushar Parekh P (2016). Cemento-ossifying fibroma of mandible mimicking complex composite odontome. BMJ Case Rep.

[10] Bala TK, Soni S, Dayal P, Ghosh I (2017). Cemento-ossifying fibroma of the mandible. A clinicopathological report. Saudi Med J.

[11] Gopinath D, Beena VT, Sugirtharaj G, Vidhyadharan K, Salmanul Faris K, Kumar SJ (2013). Cemento-ossifying fibroma in a patient with end-stage renal disease. Case Rep Dent.

